# Cerebrospinal fluid may mediate CNS ischemic injury

**DOI:** 10.1186/1743-8454-2-7

**Published:** 2005-09-20

**Authors:** Yanming F Wang, Judith K Gwathmey, Guorong Zhang, Sulpicio G Soriano, Shunli He, Yanguang Wang

**Affiliations:** 1 Neuroprotection Inc. 100 Cummings Center, Suite 439-C, Beverly, MA 01915 USA; 2 The Department of Anesthesia, Children's Hospital and Harvard Medical School, 300 Longwood Avenue, Boston, MA 02115 USA; 3 Harvard Medical School and Beth Israel Deaconess Medical Center, 330 Brookline Avenue, Boston MA 02115 USA and Gwathmey Inc. 763 Concord Avenue, Building E, Cambridge, MA 02138 USA

## Abstract

**Background:**

The central nervous system (CNS) is extremely vulnerable to ischemic injury. The details underlying this susceptibility are not completely understood. Since the CNS is surrounded by cerebrospinal fluid (CSF) that contains a low concentration of plasma protein, we examined the effect of changing the CSF in the evolution of CNS injury during ischemic insult.

**Methods:**

Lumbar spinal cord ischemia was induced in rabbits by cross-clamping the descending abdominal aorta for 1 h, 2 h or 3 h followed by 7 d of reperfusion. Prior to ischemia, rabbits were subjected to the following procedures; 1) CSF depletion, 2) CSF replenishment at 0 mmHg intracranial pressure (ICP), and 3) replacement of CSF with 8% albumin- or 1% gelatin-modified artificial CSF, respectively. Motor function of the hind limbs and histopathological changes of the spinal cord were scored. Post-ischemic microcirculation of the spinal cord was visualized by fluorescein isothiocyanate (FITC) albumin.

**Results:**

The severity of histopathological damage paralleled the neurological deficit scores. Paraplegia and associated histopathological changes were accompanied by a clear post-ischemic deficit in blood perfusion.

Spinal cord ischemia for 1 h resulted in permanent paraplegia in the control group. Depletion of the CSF significantly prevented paraplegia. CSF replenishment with the ICP reduced to 0 mmHg, did not prevent paraplegia. Replacement of CSF with albumin- or gelatin-modified artificial CSF prevented paraplegia in rabbits even when the ICP was maintained at 10–15 mmHg.

**Conclusion:**

We conclude that the presence of normal CSF may contribute to the vulnerability of the spinal cord to ischemic injury. Depletion of the CSF or replacement of the CSF with an albumin- or gelatin-modified artificial CSF can be neuroprotective.

## Background

The central nervous system (CNS) including brain and spinal cord is extremely susceptible to hypoxic-ischemic insults compared with peripheral organ systems such as the liver, kidney, lung, or intestines. The mechanism underlying this susceptibility is not completely understood. Many theories have been proposed and intensively investigated, including the involvement of oxygen free radicals, calcium overloading, excitatory amino acid release and nitric oxide [1-3]. These various mechanisms, however, have not been proven conclusively to mediate the vulnerability of the brain and spinal cord to ischemic injury.

In peripheral organ systems, capillaries are relatively permeable to proteins. The associated lymphatic system maintains lymph and interstitial fluid (ISF) protein concentration at about 2 g/dl, and ensures a negative interstitial pressure at about -3 mmHg [4]. In the CNS, the brain and spinal cord are bathed by the cerebrospinal fluid (CSF). Although the CSF system has similarities with the lymphatics, the CSF contains only about 25 mg/dl of protein [5]. In addition, intracranial pressure (ICP) averages about 10 mmHg, resulting in a positive interstitial fluid pressure [4], in contrast to the negative pressure in peripheral tissues. Hence, the CSF system may predispose the CNS to edema. Swelling of cerebral tissue can compress blood vessels inside the Virchow-Robin space leading to a persistent deficit in blood perfusion even after the restoration of blood perfusion, termed a 'no-reflow' or 'low reflow' phenomenon [6]. We therefore hypothesized that the presence of CSF and a positive ICP may explain why the CNS is more vulnerable to ischemia than peripheral organ systems.

The spinal cord has a linear contour, it is therefore possible to create a CSF free environment for the lumbar spinal cord by removing the CSF and adjusting body position. Using a rabbit model of spinal cord ischemia, this investigation distinguished the influence of the CSF from that of ICP, and demonstrated that the addition of albumin or gelatin to the CSF can prevent injury and may be neuroprotective.

## Methods

Male New Zealand white rabbits, n = 77, weighing 2.5–3 kg were used (Harlan, Indiana). Animal studies were approved by the institutional animal care and use committee (permission number 2249) and complied with the 'Principles of Laboratory Animal Care' (Guide for the Care and Use of Laboratory Animals, National Institute of Health publication 86-23, 1985).

### Pre-ischemia preparation

Each rabbit was anesthetized with isoflurane in 100% oxygen (5% for induction, 1% for maintenance) by facemask. Body temperature was maintained at 37 ± 1°C with a heating blanket. The right femoral artery was catheterized for monitoring mean arterial pressure (MAP) and for acquiring samples for blood gas analysis during surgical procedures. To remove the CSF and to monitor the ICP, a small silicon tube (OD = 0.025, ID = 0.012 inches, Braintree Scientific, MA) was placed in the cisterna magna. Briefly, an incision was made on the dorsal neck to expose the nuchal ridge and dorsal cervical musculature. The atlanto-occipital membrane was exposed by blunt dissection and pierced with a 30 gauge needle. The silicon tube was twisted gently through the hole on the membrane until CSF was noted to pulsate in the catheter. The tube was fixed in position and immobilized to the adjacent muscle by an instant adhesive cyanoacrylate gel (Plastics One, VA). To facilitate removal of the CSF and to administer albumin- and gelatin-modified artificial CSF another silicon tube was placed in the lumbar subarachnoid space. Briefly, a laminectomy was carried out at sacrococcygeal level to expose the lumbar subarachnoid space. The dura was then pierced with a 30 gauge needle. The tube was twisted gently into subarachnoid space through the dura and immobilized with the same adhesive to the adjacent muscle.

### Experimental design

Rabbits were randomly divided into 7 groups.

#### Group 1. Control group (n = 12)

No treatment was given. The ICP was monitored through the small silicon tube in the cisterna magna and maintained at 10 – 15 mmHg by withdrawing or infusing CSF through the tube in lumbar subarachnoid space. Rabbits were subjected to 1 h spinal cord ischemia.

#### Group 2. CSF depleted group (n = 12)

The objective was to provide the ischemic spinal cord with a relatively CSF free environment. Prior to spinal cord ischemia, the CSF was removed as completely as possible; usually 0.8 – 1.2 ml CSF could be withdrawn. To assist in maintaining a CSF free environment below the lumbar spinal cord, two additional proc**e**dures were used. First, while the tube in the cisterna magna was withdrawing CSF, the tube in lumbar subarachnoid space was kept open. Second, after removing the CSF, rabbits were kept in a tilted position with the head down (10 degrees) to prevent any remaining CSF from flowing to the lumbar region during the period of ischemia. After CSF depletion, rabbits were subjected to 1 h spinal cord ischemia. The ICP was monitored and maintained at 0 mmHg during the period of ischemia.

#### Group 3. CSF replenished group (n = 12)

The objective was to provide the ischemic spinal cord with a normal CSF environment while maintaining ICP at 0 mmHg. Prior to spinal cord ischemia, the CSF was removed as completely as possible as described above. To ensure that CSF was present in the lumbar spinal cord ischemic region, two procedures were used. First, 0.1 ml CSF was immediately replenished through the tube in the lumbar subarachnoid space. This volume was previously determined by withdrawing CSF *post mortem *from a rabbit weighing 2.5 kg. Second, rabbits were kept in a tilted position with head up (10 degrees) during the period of ischemia to allow any remaining CSF inside cranium to flow into the ischemic region of the spinal cord. After CSF replenishment, the rabbits were subjected to 1 h spinal cord ischemia. The ICP was monitored and maintained at 0 mmHg.

#### Group 4. Albumin-modified artificial CSF group (n = 12)

The objective of this group was to replace the CSF with albumin-modified artificial CSF in the spinal subarachnoid space. Albumin-modified artificial CSF was made by dissolving 8% bovine albumin (A6003, Sigma-Aldrich) in artificial CSF (Na^+ ^150 mEq/L, K^+ ^3.0 mEq/L, Mg^++ ^0.9 mEq/L, Ca^++ ^1.4 mEq/L, P 1.0 mEq/L, Cl^- ^155 mEq/L, glucose 60 mg/dl). Prior to spinal cord ischemia, 2 ml of albumn-modified artificial CSF was quickly flushed in through the silicon tube in the lumbar subarachnoid space and allowed to flow out through the tube in the cisterna magna. Rabbits were then subjected to 1 h spinal cord ischemia, during which time albumin-modified artificial CSF was continuously infused at rate of 2 ml/h. The ICP was monitored and maintained at 10–15 mmHg during the infusion by withdrawing or infusing the albumin-modified artificial CSF from the lumbar subarachnoid space.

#### Group 5. Gelatin-modified artificial CSF treatment group (n = 12)

The objective of this group was to replace the CSF with gelatin-modified artificial CSF in the spinal subarachnoid space and to compare it with albumin-modified artificial CSF. Gelatin-modified artificial CSF was made by dissolving 1% porcine skin gelatin (G1890, molecular weight between 50,000 – 100,000, Sigma-Aldrich) in artificial CSF. The experiment was performed as described for group 4.

#### Group 6. (n = 10)

Treatment was the same as group 2, except that all rabbits were subjected to 2 h spinal cord ischemia and the CSF removal procedure was repeated every h.

#### Group 7. (n = 7)

Treatment was the same as group 2, except that all rabbits were subjected to 3 h spinal cord ischemia and the CSF removal procedure was repeated every h.

### Induction of ischemia

After the treatment according experimental design, spinal cord ischemia was induced according to Zivin [7]. Under aseptic operative techniques, a midline abdominal laparotomy of 6–7 cm long was made and the intrarenal aorta was isolated. A Diethrich bulldog clamp (50 mm length, closing force 50 g) was used to cross-clamp the aorta just caudal to the left (lower) renal artery. In the rabbit, this corresponds to the second lumbar vertebrae (L 1–2) level. The aorta was cross-clamped for 1, 2 and 3 h according to the experimental design. Immediately after the removal of the clamp, the abdominal incision was closed in layers. After ischemia, all silicon tubes were disconnected, and the wounds were closed. Rabbits were returned to their cages, provided food and water and allowed to recover for seven days.

### Neurological deficit evaluation

After ischemic injury, each rabbit was tested daily for seven days. Motor function of the hind limbs was scored from 0 – 5 according to modified Tarlov's score [8] by an investigator blinded to the experimental group. Score 0: complete recovery, able to hop normally (the hind limbs were able to leave the ground simultaneously when hopping). Score 1: able to hop but wobbly and might fall on their side occasionally. Score 2: able to stand, but unable to hop; Score 3: good movement of the hind limbs, but unable to stand; Score 4: spastic paraplegia with slight movement of the hind limbs; Score 5: spastic paraplegia with no movement to the hind limbs. An animal with a motor function (Tarlov score) ≥ 2 was considered paraplegic.

### Histopathological evaluation

After seven days of neurological evaluation, rabbits were euthanized with an overdose of pentobarbital sodium (200 mg/kg) injection via the heart. The spinal cord was perfused with 10% buffered formalin. The spinal cords from lower thoracic level to the lower lumbar level were harvested by multiple laminectomies and fixed in a 10% buffered formalin solution for one week. Spinal cord segments at the lumbar enlargement were embedded in paraffin. Transverse sections were cut at 6 μm and stained with hematoxylin-eosin. The spinal cord lesions at L5 level were scored under the microscope at a magnification of 40× and 100× by an investigator blinded to experimental procedures according to the following criteria [9-11]:

Score 1: Number of vacuolations < 5 in grey matter of the hemi-section.

Score 2: Number of vacuolations ≥ 5 in grey matter of the hemi-section.

Score 3: The architecture of grey matter was necrotic or completely destroyed or replaced by scar tissue. No neurons could be seen.

### Visualization of the spinal cord microcirculation

At 23 h after spinal cord ischemia, lumbar spinal cord microcirculation was studied in two rabbits from groups 1–5. Each rabbit received 0.3 g/kg 15% fluorescein isothiocyante (FITC) albumin (Sigma-Aldrich) by i.v. bolus injection [12]. Rabbits were euthanized 2 min after FITC injection by the method described above. Spinal cords were immediately removed and fixed in 10% formalin overnight and transferred to 25% sucrose for 24 h. The tissues were frozen with dry ice. Transverse sections, 100 μm thick, were cut with a microtome at the level of L5, transferred to glass slides and investigated immediately by fluorescence microscopy at 40× magnification.

### Statistical analysis

The physiological parameters were analyzed with one-way repeated measures ANOVA (analysis of variance) followed by Tukey test. The neurological deficit scores and the histopathological scores were analyzed with Kruskal-Wallis ANOVA on Ranks followed by the Tukey test. A *p *value < 0.05 was considered statistically significant.

## Results

Physiological parameters at baseline and reperfusion were similar for all experimental groups and there were no significant differences between groups. In addition, there were no significant differences between baseline and reperfusion values for MAP, PO_2_, PCO_2_, pH and glucose in any group (Table 1).

**Table 1:** Physiologic parameters during rabbit spinal cord ischemia. Values are mean ± SD. Baseline physiological values were determined 5 minutes before cross-clamping the aorta. Reperfusion physiological values were determined 5 minutes after releasing the clamp around abdominal aorta. None of the physiological parameters at baseline and reperfusion differed significantly between the various experimental groups. There were no significant differences in MBP, PO_2_, PCO_2 _and glucose between baseline and reperfusion in any group. The pH value was slightly decreased after ischemia in each group, however, there were no significant differences either.

	Group						
	
	1	2	3	4	5	6	7
Variable	n = 10	n = 10	n = 10	n = 10	n = 10	n = 10	n = 7
MAP (mmHg)							
Baseline	82 ± 12	83 ± 11	86 ± 12	76 ± 7	84 ± 14	79 ± 11	81 ± 15
Reperfusion	80 ± 8	81 ± 11	81 ± 10	76 ± 11	78 ± 14	74 ± 11	73 ± 9
pH							
Baseline	7.40 ± 0.03	7.41 ± 0.03	7.40 ± 0.03	7.39 ± 0.02	7.39 ± 0.02	7.40 ± 0.03	7.40 ± 0.03
Reperfusion	7.35 ± 0.02	7.34 ± 0.03	7.35 ± 0.05	7.34 ± 0.03	7.35 ± 0.04	7.33 ± 0.02	7.33 ± 0.03
PaO_2 _(mmHg)							
Baseline	212 ± 19	221 ± 18	225 ± 24	208 ± 22	213 ± 20	226 ± 18	223 ± 24
Reperfusion	225 ± 26	209 ± 20	223 ± 20	212 ± 20	225 ± 21	230 ± 14	234 ± 31
PaCO_2 _(mmHg)							
Baseline	38 ± 3	37 ± 4	38 ± 2	37 ± 3	39 ± 2	37 ± 2	37 ± 3
Reperfusion	35 ± 7	35 ± 3	36 ± 5	35 ± 5	36 ± 3	35 ± 4	34 ± 5
Glucose (mg/dl)							
Baseline	131 ± 22	133 ± 27	123 ± 26	128 ± 29	141 ± 25	121 ± 30	126 ± 25
Reperfusion	134 ± 22	143 ± 25	117 ± 24	138 ± 30	144 ± 25	128 ± 26	135 ± 30

**Figure 1:**
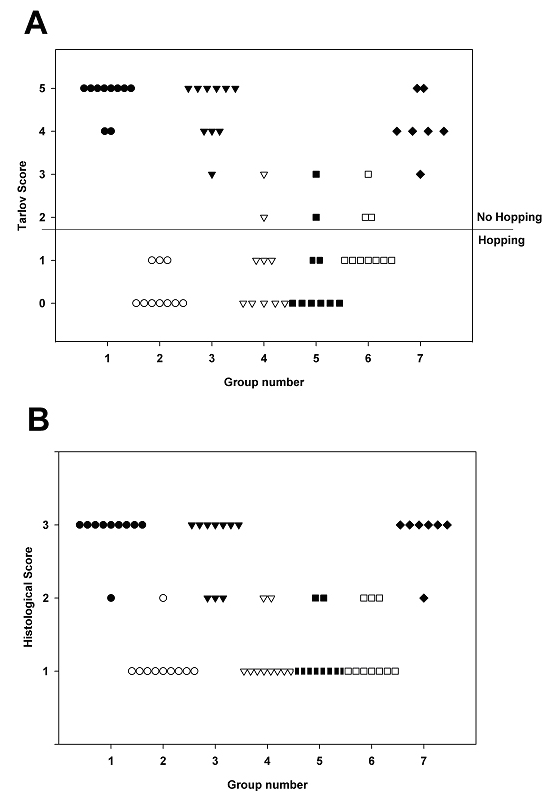
**Effect of CSF removal and replacement of CSF with albumin- and gelatine-modified artificial CSF on rabbit spinal cord ischemia**. Group 1 (closed circles). 1 h ischemia; prior treatment: none; ICP: 10–15 mmHg. Group 2 (open circles). 1 h ischemia; prior treatment: CSF depleted; ICP: 0 mmHg. Group 3 (closed triangles). 1 h ischemia; prior treatment: CSF replenished; ICP: 0 mmHg. Group 4 (open triangles). 1 h ischemia; prior treatment: 8% albumin in artificial CSF; ICP: 10–15 mmHg. Group 5 (closed squares). 1 h ischemia:; prior treatment: 1% gelatin in artificial CSF; ICP: 10–15 mmHg. Group 6 (open squares). 2 h ischemia; prior treatment: CSF depleted; ICP: 0 mmHg. Group 7 (closed diamonds). 3 h ischemia; prior treatment: CSF depleted; ICP: 0 mmHg. **A**. Neurological deficit (Tarlov score) determined at 7 d after spinal cord ischemia. Group 1 vs. groups 2, 4, 5 and 6; Group 3 vs group 2, 4 and 5; Group 7 vs. group 2 and 5 are significantly different when analyzed by Kruskal Wallis ANOVA followed by Dunn's test (*p *< 0.05). **B**. Histopathological score determined at 7 d after spinal cord ischemia by H&E staining. Groups 1, 3 and 7 vs. Groups 2, 4, 5 and 6 were significantly different analyzed by Kruskal Wallis ANOVA followed by Dunn's test (*p *< 0.05).

The neurological deficit score, taken 24 h after ischemia did not differ from the score obtained seven days after ischemia. Control animals subjected to 1 h of spinal cord ischemia demonstrated permanent paraplegia (100% paraplegic rate, Fig. 1A, group 1-*closed circles*). The CSF depletion with the ICP maintained at 0 mmHg prevented the development of paraplegia (0% paraplegic rate, Fig. 1A, group 2-*open circles*). However, replenishment of CSF around ischemic spinal cord (returning a small amount of CSF while maintaining a tilted, head-up position) did not prevent paraplegia, even when the ICP was controlled at 0 mmHg (100% paraplegic rate, Fig. 1A, group 3-*closed triangles*). Replacing the CSF with albumin- or gelatin-modified artificial CSF under physiological ICP conditions (10–15 mmHg) significantly prevented the development of paraplegia (~20% paraplegic rate, Fig. 1A, groups 4-*open triangles*, and 5-*closed squares*).

Similarly, the CSF depletion significantly prevented paraplegia in rabbits subjected to 2 h of spinal cord ischemia (~30% paraplegic rate, Fig. 1A, group 6-*open squares*), but did not significantly prevent paraplegia after 3 h of ischemia (100% paraplegic rate, Fig. 1A, group 7-*closed diamonds*).

**Figure 2:**
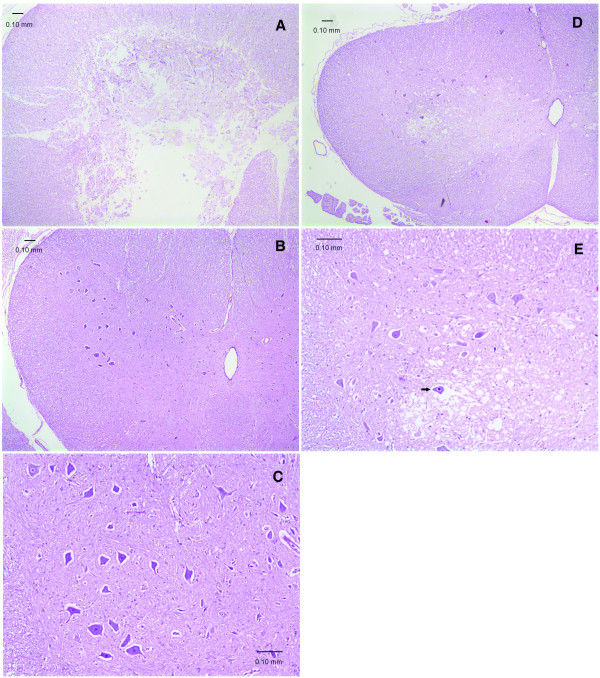
**Transverse sections of rabbit lumbar spinal cord after 1 h ischemia (H&E stain)**. **A**. Histopathological score 3, from a rabbit of Group 1. Severe necrosis destroys the entire structure of grey matter indiscriminately. **B**. Histopathological score 1, from a rabbit of Group 2. No apparent spinal cord damage, the grey matter is well preserved. **C**. Higher magnification of **B **showing normal morphology of grey matter and neurons. **D**. Histopathological score 2, from a rabbit of Group 3. The grey matter is lightly stained with many vacuolations of the neuropil. **E**. Higher magnification of **D**. Marked vacuolations of the neuropil in the grey matter, and some neurons are triangular with darkly stained shrunken nuclei (arrow).

In all groups, the scores for the severity of the histopathological damage paralleled that for the neurological deficits (Fig 1B). Typical histopathological findings following spinal cord ischemia included necrosis and vacuolation of the neuropil in the grey matter. Without CSF removal, the severe necrosis often destroyed normal architecture of the grey matter resulting in liquefaction (Fig 2A). The depletion of CSF, replacement of CSF with albumin- or gelatin-modified artificial CSF prevented signs of histopathological damage (Fig 2B&C). In contrast, the replenishment of CSF around ischemic spinal cord resulted in necrosis or severe vacuolation of grey matter (Fig 2D&E).

**Figure 3:**
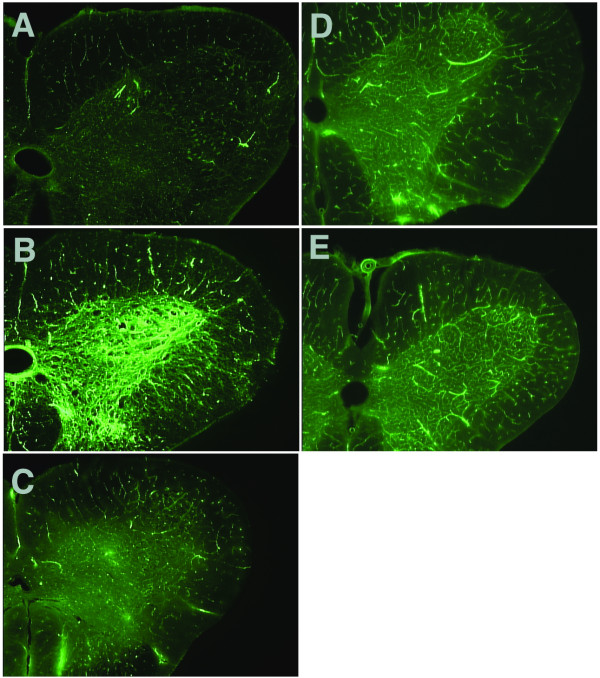
**Transverse sections of rabbit lumbar spinal cord to show the microcirculation after 1 h of ischemia and 23 h reperfusion, revealed by FITC-albumin**. **A**. No CSF removed (Group 1) – the spinal cord demonstrates extremely faint fluorescein signal with absence of capillary filling in both grey matter and white matter, indicating a 'no-reflow' phenomenon. **B**. Depletion of the CSF (Group 2) – spinal cord section demonstrates intensive fluorescein signals, which are much stronger in grey matter than in white matter, indicating good blood perfusion. **C**. Replenishment of the CSF (Group 3) – grey matter demonstrates faint fluorescein signal similar to white matter, indicating a marked blood perfusion deficit, i.e. 'low-reflow' phenomenon. **D**. Albumin-modified artificial CSF replacement (Group 4), and **E**. Gelatin-modified artificial CSF replacement (Group 5) – capillary filling in both grey matter and white matter are clearly demonstrated by good fluorescein signal, albeit slightly less than that of Group 2, indicating some preservation of blood perfusion.

Normal microcirculation of the spinal cord when visualized by FITC-albumin demonstrated good capillary filling with clear contrasting of fluorescein signals between grey and white matter (stronger in grey matter than in white matter). After ischemia the 'no-reflow' phenomenon was characterized by the absence of capillary filling in both the grey matter and white matter. A 'no-reflow' phenomenon of lumbar spinal cord was demonstrated in control rabbits (Fig 3A). In contrast, normal microcirculation of lumbar spinal cord was observed after ischemia in rabbits with the depletion of CSF, or replacement of CSF by albumin- or gelatin-modified artificial CSF (Fig 3B,D&E). The 'low-reflow' phenomenon was also noticed in rabbits with the replenishment of CSF (Fig 3C).

## Discussion

The CNS lacks a lymphatic system; instead it is surrounded by the CSF. The CSF is very different from the lymph in peripheral tissues in at least two aspects: protein concentration and the resultant interstitial fluid pressure.

In peripheral tissues, capillaries are relatively permeable and as a result the ISF contains about 2 g/dl of plasma proteins. It is believed that interstitial proteins and hyaluronic acids form a dense network of proteoglycan filaments that impede fluid flow through the interstitium. Normally the amount of free-flowing fluid, present in the interstitium is small. A low interstitial protein concentration results in an increased amount of free ISF. An elevated concentration of interstitial protein may reduce the free ISF, but it also attracts more fluid, resulting in increased volume. The lymphatic system is the scavenging pathway for interstitial proteins. By regulating the removal of excess protein, the lymphatic system keeps the interstitial protein concentration around 2 g/dl. This ensures limited free fluid and also regulates the ISF volume. Lymph flow reduces ISF volume resulting in negative interstitial pressure. Therefore, the movement of proteins from plasma to ISF and finally to lymph is important for maintaining extracellular homeostasis.

**Figure 4:**
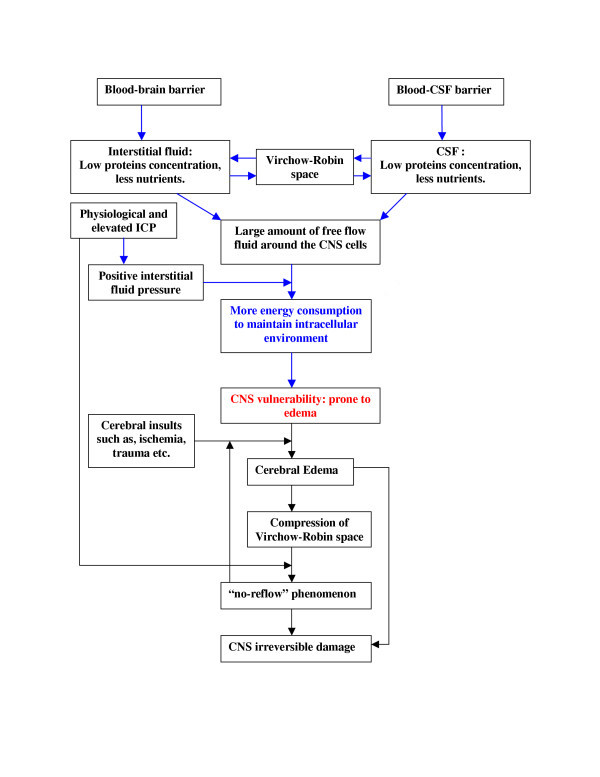
**Overview of mechanisms underlying the CNS vulnerability to ischemia**. Blood brain barrier and Blood-CSF barrier determine the low protein concentration in the cerebrospinal fluid and the interstitial fluid. A large amount of free fluid causes the CNS cells to consume more energy to maintain normal intracellular environment. The positive intracranial pressure facilitates the edema formation. Edema results in 'no-reflow' phenomenon.

In the CNS, the CSF is secreted by the choroid plexuses that line the cerebral ventricles. Tight junctions linking the adjacent choroidal epithelial cells form the blood-CSF barrier and prevent most large molecules from passing into the CSF from the blood. Therefore the CSF contains an extremely low protein concentration. The choroid plexuses may not be the only sites for CSF production. Milhorat reported that in monkeys with choroid plexuses removed, up to 60% of the CSF is produced from ISF flow out of the brain [13]. The ISF has a bulk flow rate of 0.1–0.3 μl/min/g in rat brain along preferential pathways especially the Virchow-Robin spaces and axon tracts [14]. Formation of ISF is thought to occur by active transport processes at the cerebral capillary [15]. The blood-brain barrier (BBB) prevents proteins from entering the interstitium. Therefore, it is speculated that the ISF in brain, just like the CSF, has a low protein concentration. It is estimated that intracellular protein concentration averages about 16 g/dl in mammalian cells [4]. Therefore water and Na^+ ^in the ISF have the potential to move easily into cells. More importantly, the CSF is contiguous with the ISF, with the Virchow-Robin spaces, serving as a conduit. To make matters worse, the ICP averages about 10 mmHg leading to a positive interstitial fluid pressure. Taken together, these factors make the CNS prone to edema formation. As a result cells in the CNS constantly consume energy to remove excess intracellular fluid in physiological condition. When cell energy is compromised, such as in ischemia, or when the cell membrane is damaged by direct trauma, cells rapidly become swollen, i.e. cytotoxic edema (Fig 4).

By taking advantage of the linear contour of the spinal cord, we were able to verify the role of CSF independently of the influence of ICP. All rabbits in the control group developed permanent paraplegia, necrosis of grey matter and associated 'no-reflow' phenomenon. Depletion of the CSF around the ischemic spinal cord significantly prevented paraplegia and associated pathological changes (group 2). Depletion of the CSF creates a partially CSF free environment in the ischemic spinal cord region. Although depletion of the CSF does not eliminate ISF, it limits the extent to which the ISF can be replenished from the CSF and limits occurrence of edema. It is estimated that the total volume of CSF is about 2.3 ml or 0.67 ml/kg for an adult rabbit, and the CSF production rate is reportedly to be between 7.8–8.8 μl/minute [16-18]. We were only able to remove 0.8–1.2 ml CSF. We believe that the remainder of the CSF may reside inside the cranium as we took several steps to approximate the CSF free environment in the ischemic spinal cord region. The depletion of CSF also reduced the ICP to 0 mmHg. Therefore, in group three, we tested the presence of the CSF around the ischemic spinal cord while maintaining a 0 mmHg ICP. This allowed us to examine the role of CSF without the influence of ICP. The data demonstrated that the CSF itself is detrimental to ischemic spinal cord.

Using albumin-modified artificial CSF to replace the CSF significantly prevented paraplegia even when the ICP was maintained within the physiological range. These data indicate that detrimental effect of the CSF may be largely ascribed to its low protein concentration. A high concentration of protein present in artificial CSF not only provides limited free fluid to ISF, but may also increase the protein concentration in ISF resulting in reduced amount of the free fluid in interstitium. The increase of protein concentration in ISF was less likely to result in volume expansion in the interstitium because of the protein concentration gradient between the CSF and the ISF; any ISF volume increase would be transferred to the protein-modified artificial CSF. We believe that the ultimate reason why protein is important for the extracellular environment may be due chiefly to its water and ion binding capacity. The water binding capacity for albumin is so large that it is estimated that one gram of albumin can bind 18 ml of water [19,20]. A number of physiological functions have been reported for albumin, such as an anti-inflammatory effect [21,22]. However, these alternative functions are less likely to be the cause of the beneficial effect because gelatin, which is mainly comprised of denatured proteins, was equally neuroprotective. Although the albumin and gelatin are both colloid osmotic agents, their efficacies in preventing the spinal cord damage were not likely to be due to their molecular weight, as we found that other colloid osmotic agents, such as 10% Dextran (molecular weight 40, 000–70,000 daltons) and 6% Hetastarch (molecular weight 400,000–550,000 daltons) which were known to create similar colloidal osmotic pressure were ineffective (Y. Wang, unpublished observations). In addition to low protein concentration, many nutrients are also lower in the CSF. For examples, the CSF contains about two third of plasma glucose concentration (CSF: 61 mg/dl; plasma: 92 mg/dl), and it contains about one fifteenth of plasma insulin concentration (CSF: 4 μU/ml; plasma: 20–30 μU/ml) [23-25]. Harkness and co-workers showed that the adenosine 5'-triphosphate (ATP) concentration was about 1 to 20 μmol/l in plasma, however, it was not measurable in the CSF [26]. Muñoz and co-workers reported that the ATP concentration in CSF is only about 16 nM/l [27]. Therefore, additional studies are needed to examine if the deficiency of these nutrients in normal CSF or the presence of any other microconstituents in CSF, may also contribute to vulnerability of the CNS.

Rabbits with albumin- or gelatin-modified artificial CSF replacement maintained with a physiological ICP still showed ~20% paraplegic rate, while depletion of the CSF showed 0% paraplegic rate, although the difference was not statistically significant by Fisher exact test. The effect of depleting the CSF may be partially due to ICP reduction, and the 20% paraplegic rate may be explained by the presence of physiological ICP. Elevated ICP has been known to be clinically detrimental. For example, CSF drainage has been used for almost 50 years to prevent paraplegia during aortic surgery when the aorta needs to be cross-clamped [28,29]. While this approach has been reported to be effective in many studies [30-38], in some examples positive outcomes were not achieved [39-41]. Crawford et al performed a randomized clinical study using CSF drainage in 98 patients that underwent cross-clamping of the aorta during surgery [39]. They controlled the ICP at 10–15 mmHg while draining the CSF, and did not find this approach effective in preventing paraplegia. We believe that this negative outcome might be attributed to the normal level of ICP and a large amount of CSF remaining in the ischemic areas of the spinal cord. These clinical outcomes in conjunction with our studies indicate that both the presence of CSF and physiological ICP are detrimental to ischemic spinal cord. The presence of CSF may be a fundamental issue.

Our experiments demonstrated a clear 'no-reflow' and 'low-reflow' phenomenon, which was accompanied by paraplegia and histopathological damage in the spinal cord. Depletion of the CSF or replacement with albumin- or gelatin-modified artificial CSF prevented the 'no-reflow' phenomenon, associated motor function deficit and histopathological spinal cord damage. Although many investigators reported blood perfusion deficits following brain and spinal cord injuries [42-44], the mechanism was not clear. Our findings suggest that this post-ischemia deficit in blood perfusion may be linked, at least in part, to the CSF associated edema and collapsing of blood vessels in the Vichow-Robin space. This blood perfusion deficit likely blocks collateral circulation and induces a feedback loop contributing irreversible cell death and tissue necrosis (Fig 4).

It has been shown that just 25 minutes of spinal cord ischemia in this rabbit model is enough to cause permanent paraplegia [10,15]. In our experiments, depletion of the CSF prevented spinal cord damage after 1 h of ischemia. With the extension of the ischemic period to 2 h, there was still significant protection of the spinal cord (group 6). Permanent damage of the spinal cord was only seen after 3 h of ischemia (group 7). These results indicate that a greater level of resistance to ischemic injury can be conferred to the spinal cord in a transiently CSF-free environment.

## Conclusion

In summary, our findings suggest that the extracellular environment, consisting of a low protein concentration in the CSF and the ISF and a positive interstitial pressure, may determine the vulnerability of CNS to ischemia. This study has determined that depletion of the CSF or replacing the CSF with albumin-, or gelatin-modified artificial CSF in combination with a lowered ICP, provides protection in the setting of ischemic injury to the CNS.

## Competing interests

The author(s) declare that they have no competing interests.

## Authors' contributions

YFW: conceived of the study, designed and coordinated all experiments, participated in spinal cord ischemia experiment in rabbits and drafted the manuscript.

JKG: helped in the preparation of the manuscript and data analysis.

GZ: participated in spinal cord ischemia experiment in rabbits.

SGS: participated in data analysis and helped in the revision of the manuscript.

SH: participated in spinal cord ischemia experiment in rabbits.

YW: participated in spinal cord ischemia experiment in rabbits.

All authors have read and approved the final manuscript.
